# Long Noncoding RNA H19 Participates in the Regulation of Adipose-Derived Stem Cells Cartilage Differentiation

**DOI:** 10.1155/2019/2139814

**Published:** 2019-05-05

**Authors:** Hai-lin Pang, Qian-qian Zhao, Yu Ma, Yong-li Song, Jie Min, Jian-rong Lu, Hang Li, Da-qing Zhao

**Affiliations:** ^1^Department of Otolaryngology, Tangdu Hospital, The Air Force Medical University, 569 Xinsi Road, Xi'an 710038, China; ^2^Department of Oncology, Tangdu Hospital, The Air Force Medical University, 569 Xinsi Road, Xi'an 710038, China; ^3^State Key Laboratory of Cancer Biology and Department of Pathology, Xijing Hospital, The Air Force Medical University, 127 Changle Western Road, Xi'an 710032, China; ^4^Department of Otolaryngology, Xijing Hospital, The Air Force Medical University, 127 Changle Western Road, Xi'an 710032, China

## Abstract

Adipose-derived stem cells (ADSCs) are multipotent and have received increasing attention for their applications in medicine. Cell-based therapies are optimal for diseases with loss or damage to tissues or organs. ADSCs and bone marrow mesenchymal stem cells (BMSCs) can differentiate into many cell lineages. Because of their advantages in accessibility and volume, ADSCs are regarded as a desirable alternative to BMSCs. In this study, we focused on the chondrocytic differentiation potential of ADSCs and the underlying mechanism. We found that the long noncoding RNA H19 plays an important role in this process. Overexpression of H19 in ADSCs induced differentiation towards chondrocytes. H19 is abundantly expressed during embryonic development and downregulated after birth, implying its regulatory role in determining cell fate. However, in our experiments, H19 exerted its regulatory function during cartilage differentiation of ADSCs through competing miRNA regulation of STAT2.

## 1. Introduction

Mesenchymal stem cells (MSCs) have promising application in various diseases, especially in regenerative medicine, because of their multipotent differentiation potential. According to the different tissue sources, MSCs can be classified into two types: bone marrow stem cells (BMSCs) and adipose-derived stem cells (ADSCs) [1]. BMSCs became well known by the end of the 1990s for their ability to propagate under tissue culture conditions and to differentiate *in vitro* into osteogenic cells [2, 3]. This regenerative ability favored their use in bone tissue engineering [4, 5]. However, difficulties in accessibility and insufficient numbers limit the use of BMSCs. An alternative source of MSCs was thus needed. Multiple independent studies have demonstrated the multilineage differentiation capacity of ADSCs. Their bone regeneration potential has been studied *in vitro* and *in vivo* both in animal models and in clinical trials [6–9]. As a by-product of liposuction procedures, ADSCs can be isolated from lipoaspirate with a relative high output. Compared with BMSCs, there is no risk of iatrogenic deformity and donor-site morbidity by using ADSCs [10, 11], though the osteogenic potential of ADSCs is inferior to that of MSCs obtained from bone marrow [12, 13]. The easily accessible and abundant nature of ADSCs are advantageous and had generated interest in their potential clinical use [8, 9, 14].

Under lineage-specific conditions, ADSCs have the capability to differentiate into cells of mesodermal origin, such as adipocytes, fibroblasts, myocytes, osteoblasts, and chondrocytes. Additionally, ADSCs have been demonstrated to transdifferentiate into nonmesodermal cell types like endothelial cells, hepatocytes, neuronal cells, and cardiomyocytes [15]. Based on these findings, ADSCs have been applied in clinical settings, predominantly to treat osteoarthritis (OA) or bone injury [16]. OA is a cartilage degenerative process with limited symptomatic treatment [17]. Specifically, a series of factors damages chondroblasts, chondrocytes, and their secretions that form the extracellular matrix (ECM), resulting in a loss of structure and function [18, 19]. Patients with OA suffer from long-term pain, and arthritis is the most common cause of disability in the USA [20]. The therapeutic potential of ADSCs for cartilage regeneration holds promise in bone diseases and reconstructive surgery. The application of ADSCs in osteoarthritis, alone and combined with other treatments, has been reported in numerous studies; the administration of ADSCs is typically followed by a significant improvement in symptoms such as reduced pain and improved range of motion and joint structure [21–25]. The underlying rationale for the use of ADSCs is their ability to differentiate into chondrocytes under appropriate conditions, as observed in 3D culture [6, 26–29].

Accumulating data demonstrates that long noncoding RNAs (lncRNAs) are involved in the differentiation and maintenance of stem cells by interacting with correlated transcription factors [30]. LncRNA H19 was the first lncRNA discovered; it is paternally imprinted [31] and has been reported to be associated with the differentiation of human articular chondrocytes [32, 33]. In this study, we induced the differentiation of ADSCs into chondrocytes and found that H19 was involved, and overexpression of H19 promoted the differentiation of ADSCs into chondrocytes. We then used the KEGG database to screen for differentially expressed genes (DEGs) during this process as well as the underlying candidate pathways. Finally, we validated these differentially expressed genes by RT-PCR and western blot. We speculate that H19 exerts its function by regulating the transcription of related genes by interacting with certain miRNAs. Understanding the molecular mechanisms of the differentiation process is essential to further the use of ADSCs in medicine.

## 2. Materials and Methods

### 2.1. Cell Culture

Third-generation ADSCs were a kind gift from the State Key Laboratory of Military Stomatology, Center for Tissue Engineering, School of Stomatology, Fourth Military Medical University, Xi'an, 710032, China, and the immunophenotype and multilineage differentiation ability were previously identified [34].ADSCs were cultured in DMEM (C11995500bt, Gibco, Gaithersburg, MD, USA) supplemented with FBS (10%; S4115, Biochrom GmbH, Berlin, Germany). Cartilage-inducing ADSCs were supplemented with ITS+1 Liquid Media Supplement (1 : 100; I2521, Sigma-Aldrich; Merck KGaA, Darmstadt, Germany), TGF-*β*1 (10 ng/mL; 5231LF, Cell Signaling Technology), FGF (25 ng/mL; 341590, Merck), and dexamethasone (0.1 *μ*M; 265005, Merck) in the above medium to the 6th generation. All cells were cultured with 100 U/mL streptomycin and 100 U/mL penicillin at 37°C with 5% CO_2_.

### 2.2. Histological Staining

Cells were cultured on coverslips (J24001, JingAn Biology, Shanghai, China) in 24-well plates and fixed with 4% paraformaldehyde. HE staining, Alcian blue staining, Masson staining, and Alizarin red staining were completed in the Department of Pathology, Air Force Medical University.

### 2.3. Immunocytochemistry

Cells in log phase were harvested and plated into an immunocytochemistry chamber. After overnight culture at 37°C for adhesion, the medium was removed and fixed with 4% paraformaldehyde. The cells were probed with monoclonal rabbit anti-collagen II (1 : 100; ab34712, Abcam) and control IgG overnight at 4°C. After washing, Histostain™-SP Kits (SPN-9001; Zhongshan Jinqiao, Beijing, China) were used following the protocol. Cells were DAB and hematoxylin stained and imaged under a microscope (IX53, Olympus).

### 2.4. Reverse Transcription-Quantitative Polymerase Chain Reaction (RT-qPCR)

Total RNA was isolated from cells using TRIzol reagent (Thermo Fisher Scientific, Inc.) following the manufacturer's instructions. Consequently, RNA (500 ng) was reverse-transcribed into cDNA by a QuantiNova™ Reverse Transcription kit (QIAGEN GmbH, Hilden, Germany). qPCR was performed using SYBR Premix Ex Taq™ II (Takara Bio, Inc., Otsu, Japan). The thermocycling conditions were as follows: denaturation at 95°C for 30 s, followed by 40 cycles at 95°C for 5 s, and 60°C for 30 s. Real-time fluorescence signals were detected with the Agilent Mx3005P qPCR System (Agilent Technologies, Inc., Santa Clara, USA), and gene expression was quantified using the 2^−ΔΔCq^ method.

### 2.5. Cell Stable Transfection

H19-overexpressing plasmids and empty vector pcDNA3.1 were obtained from Shanghai GenePharma Co., Ltd, Inc. 1.2 *μ*g plasmids were transfected into ADSCs in 6-well plates with Lipofectamine 2000 (Invitrogen; Thermo Fisher Scientific, Inc.) according to the manufacturer's instructions. A total of 48 h after transfection, cells were harvested for subsequent experiments.

### 2.6. RNA Sample Preparation for RNA Sequencing

Total RNA was extracted from ADSC-H19 and control ADSC-pcDNA3.1 cells by TRIzol reagent (Invitrogen) separately. The RNA quality was checked by a Bioanalyzer 2200 (Agilent) and kept at −80°C. miRNA (RIN > 6.0) was purified by an miRNeasy Mini Kit (Qiagen), and the purity was validated by gel electrophoresis.

#### 2.6.1. cDNA Library Construction

cDNA libraries were constructed for each pooled RNA sample using the NEBNext® Ultra™ Directional RNA Library Prep Kit for Illumina according to the manufacturer's instructions. Generally, the protocol consists of the following steps: depletion of rRNA and fragmentation into 150–200 bp segments using divalent cations at 94°C for 8 min. The cleaved RNA fragments were reverse-transcribed into first-strand cDNA, followed by second-strand cDNA synthesis, and fragments were end-repaired, A-tailed, and ligated with indexed adapters. Target bands were harvested using AMPure XP Beads (Beckman Coulter). The products were purified and enriched by PCR to create the final cDNA libraries and quantified by an Agilent 2200. The tagged cDNA libraries were pooled in equal ratios and used for 150 bp paired-end sequencing in a single lane of an Illumina HiSeq 4000.

### 2.7. miRNA Library Construction and RNA Sequencing

The cDNA libraries for single-end sequencing were prepared using an Ion Total RNA-Seq Kit v2.0 (Life Technologies) according to the manufacturer's instructions. cDNA libraries were size-selected by PAGE for miRNA sequencing, then processed for the proton sequencing process according to the commercially available protocols. Samples were diluted and mixed, and the mixture was processed on a OneTouch 2 instrument (Life Technologies) and enriched on a OneTouch 2 ES station (Life Technologies) for preparing the template-positive Ion PI™ Ion Sphere™ Particles (Life Technologies) according to Ion PI™ Hi-Q OT2 200 Kit (Life Technologies). After enrichment, the mixed template-positive Ion PI™ Ion Sphere™ Particles of samples was loaded onto one v3 Proton Chip (Life Technologies) and sequenced on a proton sequencer with an Ion PI Hi-Q Sequencing 200 Kit (Life Technologies) by NovelBio Corp. Laboratory, Shanghai.

### 2.8. Western Blot Analysis

Cellular protein was extracted by RIPA buffer (Beyotime Biotechnology, Shanghai, China). After quantification with a BCA kit (Pierce, Thermo Fisher Scientific, Inc.), 20 *μ*g of protein was subjected to 10% SDS-PAGE and transferred to nitrocellulose membranes. The members were then blocked in 5% fat-free milk for 1 h at room temperature and incubated overnight at 4°C with rabbit anti-collagen II (1 : 1000; ab188570; Abcam), rabbit anti-Aggrecan (1 : 6000; 13880-1-AP; ProteinTech Group, Inc.), rabbit anti-Stat2 polyclonal antibody (1 : 400; GTX59884; GeneTex, Irvine, CA, USA) or rabbit anti-IRF9 polyclonal antibody (1 : 1000; PA5-40357, Thermo Fisher Scientific, Inc. Rockford, IL, USA), or *β*-actin (1 : 10,000; 051M4892; Sigma-Aldrich, Merck KGaA, Darmstadt, Germany). Next, peroxidase-conjugated goat anti-rabbit IgG (1 : 5,000; ZB-2301; Zhongshan Jinqiao) or peroxidase-conjugated goat anti-mouse IgG (1 : 5,000; ZB-2305; Zhongshan Jinqiao) were incubated for 1 h at room temperature. Finally, the target proteins were visualized by chemiluminescence (Pierce, Thermo Fisher Scientific, Inc.).

### 2.9. Statistical Analysis

Statistical tests were performed with GraphPad Prism 6 (GraphPad Software, Inc., La Jolla, CA, USA). Data are presented as the mean ± SEM, and comparisons between 2 groups were tested by Student's unpaired *t*-test. *P* < 0.05 was considered statistically significant.

## 3. Results

### 3.1. ADSC Cartilage Induction

**Figure 1:**
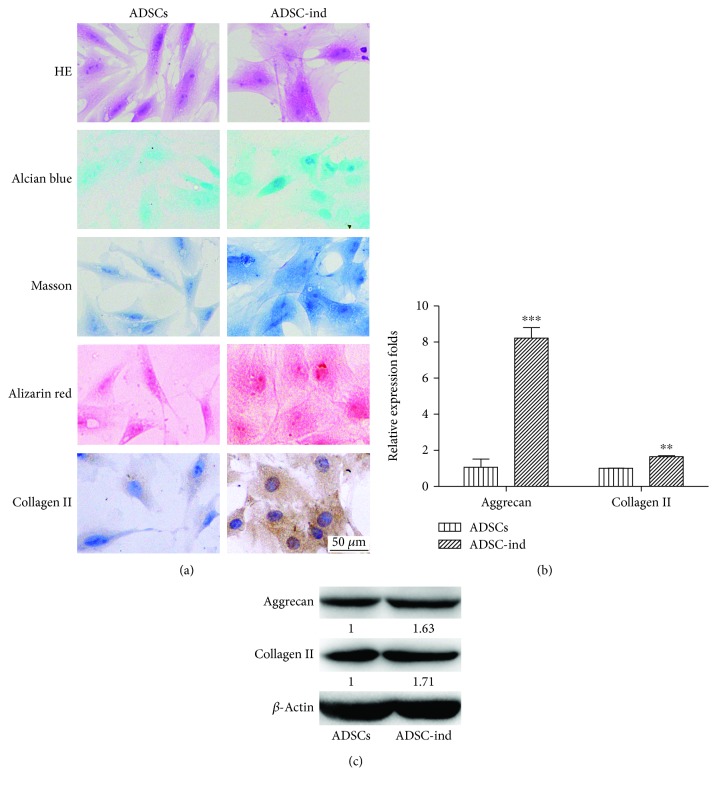
The ADSCs were induced to cartilage differentiation. (a) Histological staining of ADSCs and cartilage-induced ADSCs (200x). (b) The mRNA level of aggrecan and collagen II in ADSCs and cartilage-induced ADSCs. (c) Protein levels of aggrecan and collagen II were confirmed using western blot (^∗∗^ *P* < 0.01, ^∗∗∗^ *P* < 0.001).

ADSCs were cultured with cartilage-inducing culture medium to the sixth generation (about 2 weeks), and histological staining was performed to probe the characteristics of cartilage cells. After cartilage-inducing culture, the cell proliferation rate and volume increased significantly and the shape changed from long fusiform into large, multi-polygonal pseudopodia. Nuclei were also larger, and nucleoli were clear; an acid mucopolysaccharide component (green) was detected in induced chondrogenic cells by Alcian blue staining, small red calcium nodules were discovered by Alizarin red staining, and collagen was more highly expressed after cartilage induction according to Masson staining and immunocytochemistry (Figure 1(a)).

To further quantify the level of chondrogenesis, the cartilage markers including aggrecan and collagen II were detected by RT-qPCR and western blot. The protein and mRNA expression levels of aggrecan and collagen II in ADSCs induced were higher than those in the control group's ADSCs (Figures 1(b) and 1(c); *P* < 0.001 or *P* < 0.01).

### 3.2. Expression Levels of Long Noncoding RNA H19 in Cartilage-Induced ADSCs and ADSC-H19

**Figure 2:**
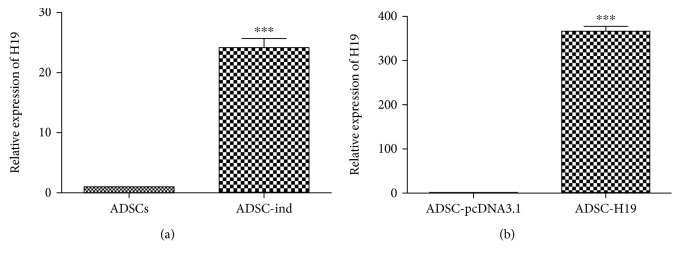
Expression of H19 in cartilage-induced ADSCs and H19-overexpressing ADSCs. (a) The expression of H19 in cartilage-induced ADSCs and parental ADSCs. (b) The expression of H19 in ADSC-H19 and control ADSC-pcDNA3.1 cells (^∗∗∗^ *P* < 0.001).

We used RT-qPCR to detect the expression of H19 in cartilage-induced ADSCs, and the expression of H19 (Figure 2(a)) was clearly increased (23.15 ± 1.534) after cartilage-inducing culture (*P* = 0.0001). We then constructed an H19-overexpressing ADSC cell line (ADSC-H19), and transfection efficacy of H19 in ADSCs was analyzed by RT-qPCR. Briefly, H19 (Figure 2(b)) expression levels in ADSC-H19 were stably increased (366.6 ± 11.02) compared with the control group ADSC-pcDNA3.1 (*P* < 0.0001).

### 3.3. H19 Induced ADSC Cartilage Differentiation

**Figure 3:**
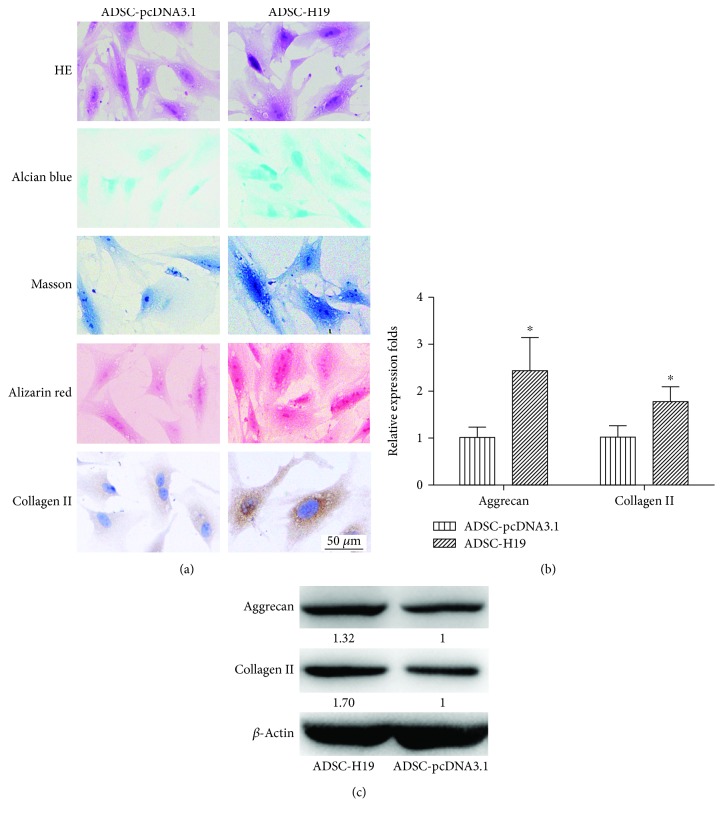
H19-overexpressing ADSCs tended to cartilage differentiation. (a) Histological staining of H19-overexpressing ADSCs (200x). (b) The mRNA level of aggrecan and collagen II in ADSC-H19 and control ADSC-pcDNA3.1. (c) Protein levels of aggrecan and collagen II were confirmed using western blot (^∗^ *P* < 0.05).

When we upregulated H19 expression in ADSCs, they tended toward cartilage differentiation. Compared with the control group ADSC-pcDNA3.1, the ADSC-H19 cell proliferation rate and volume increased significantly. The morphological characteristics were similar to those induced by cartilage induction medium as shown in Figure 1. The shape changed from long fusiform to multipolygonal pseudopodia; the nuclei were larger; nucleoli were clear; and staining and immunocytochemistry indicated the presence of acid mucopolysaccharide, calcium nodules, and high levels of collagen expression (Figures 3(a) and 3(c)).

To further quantify the level of chondrogensis, the cartilage markers including aggrecan and collagen II were detected by RT-qPCR and western blot. The protein and mRNA expression levels of aggrecan and collagen II in induced ADSCs were higher than those in the control group ADSCs ((Figures 3(b) and 3(c); *P* < 0.05).

### 3.4. Differential Expression in Pathway Analysis

**Figure 4:**
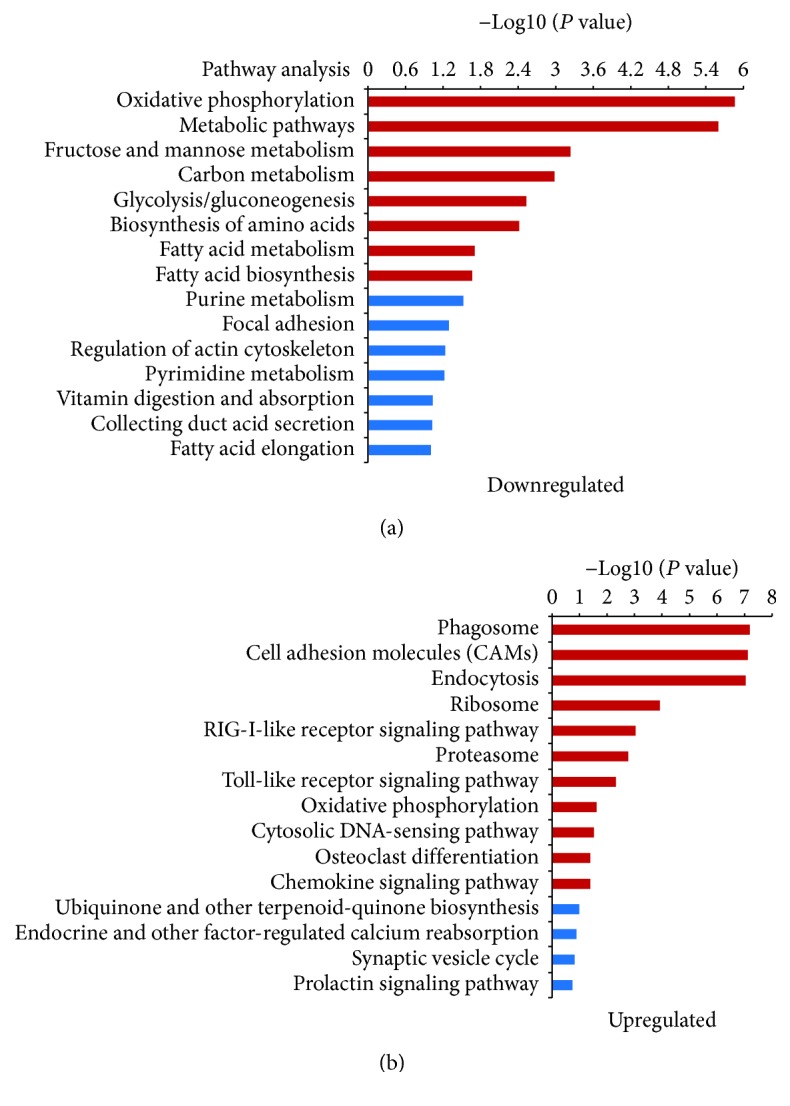
Pathway enrichment analysis of DEGs between ADSC-H19 and control ADSC-pcDNA3.1 cells. (a) Clustering of downregulated genes involved in the pathway enrichment analysis of H19 overexpression cells. (b) Clustering of upregulated genes involved in the pathway enrichment analysis of H19 overexpression cells. (Red bands represent genes in this pathway were significantly differentially expressed (FDR < 0.05 and *P* < 0.05), and green bands represent DEGs (FDR > 0.05 or *P* > 0.05).)

To probe the mechanism of H19 involved in cartilage differentiation, we applied RNA sequencing in our study, and the KEGG database was used to annotate the screened differential expressed genes (DEGs). According to RNA sequencing and results analysis, a total of 276 DEGs were obtained (*P* < 0.05 and FDR < 0.05), and 113 genes were included in the pathway category analysis. Some pathway terms related to fatty acid metabolism exhibited significant inhibition, such as fatty acid biosynthesis and the glycolysis/gluconeogenesis signaling pathway, as well as metabolic processes (oxidative phosphorylation, metabolic pathways, fructose and mannose metabolism, carbon metabolism, and purine metabolism) (Figure 4(a)). Many more pathway terms that exhibited significant upregulation included several major processes, such as biosynthesis processing (the ribosome signaling pathway, the proteasome signaling pathway, the oxidative phosphorylation signaling pathway, and cytosolic DNA-sensing pathway), cell adhesion molecules, the chemokine signaling pathway, the RIG-I-like receptor signaling pathway, the osteoclast differentiation pathway, and immune system processes (Toll-like receptor signaling pathway) (Figure 4(b)).

### 3.5. The H19 Differentially Expressed Gene Interaction Network

**Figure 5:**
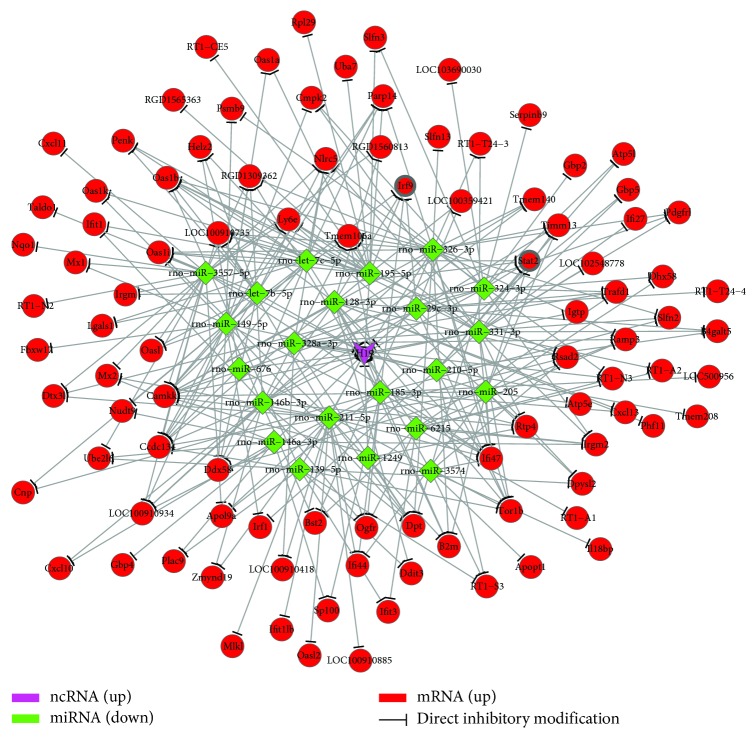
H19 participates in the regulatory differentially expressed gene interaction network via ceRNA-mediated mRNA expression.

To elucidate the association between H19 and ADSCs cartilage differentiation, H19, miRNAs, and mRNAs identified in our analysis were used to establish a ceRNA (competing endogenous RNA) network based on the KEGG database to determine the associations between the identified DEGs (Figure 5). The ceRNA analysis showed that 22 miRNAs that directly inhibited the expression of H19 were downregulated; and furthermore, 97 mRNAs inhibited by these miRNAs were upregulated. Consistent with the pathway analysis results, STAT1, STAT2, IRF9, Dhx58, Ddx58, Cxcl10, Cxcl11, and Cxcl13, which were previously identified in our pathway analyses (osteoclast differentiation pathway and/or chemokine signaling pathway and/or RIG-I-like receptor signaling pathway), appeared to be very important genes for cartilage differentiation according to the ceRNA network analysis, because these genes exhibited a stronger degree of centrality; and the three signaling pathway-related genes were upregulated and interacted with each other. Furthermore, miR-185-3p, miR-128-3p, miR-149-5p, miR-29c-3p, miR-195-5p, let-7c-5p, let-7b-5p, miR-324-3p, miR-205, and miR-210-5p showed interactive effects with many upregulated differentiation-associated genes.

### 3.6. Coexpression of H19, STAT2, and IRF9 in H19-Overexpressing ADSCs, Validation of Representative Genes Using qRT-PCR and Western Blot, and Construction of an Interactive Network

**Figure 6:**
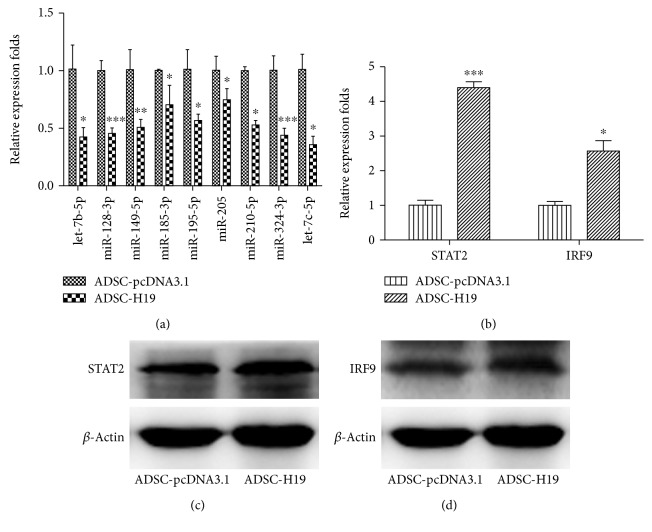
Validation of relative expression levels of representative DEGs in the osteoclast differentiation pathway. (a) Representative 9 miRNAs detected by RNA-Seq were confirmed using qRT-PCR. (b) The mRNA levels of STAT2 and IRF9 detected by RNA-Seq were confirmed using qRT-PCR. (c) Protein levels of STAT2 were confirmed using western blot. (d) Protein levels of IRF9 were confirmed using western blot (^∗^ *P* < 0.05, ^∗∗^ *P* < 0.01, ^∗∗∗^ *P* < 0.001).

**Figure 7:**
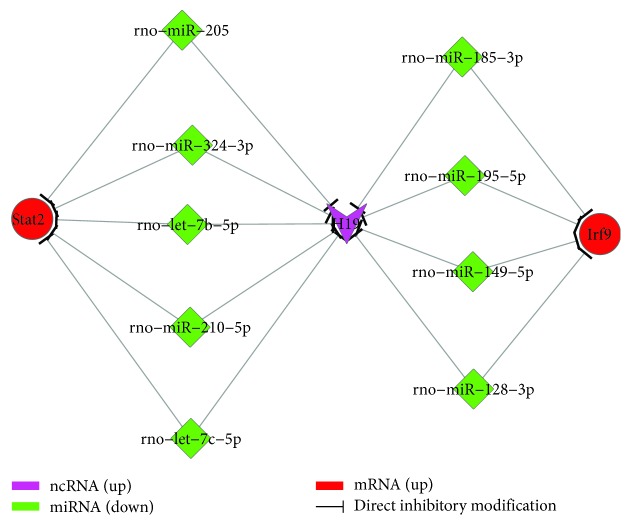
H19-miRNA-STAT2/IRF9 interactive network in ADSC cartilage differentiation.

To further validate the accuracy of our sequencing results, an H19-overexpressing ADSC cell line was established as described previously, and the expressions of osteoclast differentiation pathway-related genes were detected using qRT-PCR (Figure 6(b)) and western blot (Figures 6(c) and 6(d); related miRNAs were also detected using qRT-PCR (Figure 6(a)). STAT2 and IRF9 were expressed at higher levels in the ADSC-H19 cell line than in control ADSC-pcDNA3.1 cells, while miR-185-3p, miR-128-3p, miR-149-5p, miR-195-5p, let-7c-5p, let-7b-5p, miR-324-3p, miR-205, and miR-210-5p were downregulated (*P* < 0.05), which was consistent with our sequencing results. We then constructed a coexpression ceRNA network based on the KEGG database, our RNA sequence data, and validated results (Figure 7). The results showed that H19 overexpression was positively correlated with the expression of STAT2 and IRF9 and negatively correlated with the expression of miR-185-3p and others. H19 may participate in the regulation ADSC cartilage differentiation via the ceRNA-mediated STAT2 pathway.

## 4. Discussion

Long noncoding RNAs (lncRNAs) represent a group of noncoding RNAs that are over 200 nucleotides in length with multiple functionalities that are not translated into polypeptides. They are involved in diverse physiological and pathological processes [35, 36]. In this study, we tried to induce the differentiation of chondrocytes from ADSCs. Compared with the multipotent ADSCs, ADSC-induced chondrocytes were larger, the cells were transformed from long fusiform shapes into polygons, and the nuclei were clearer and larger. The expressions of acid mucopolysaccharide, calcium nodules, and collagen were obviously upregulated in the ADSC-induced chondrocytes, confirming their identity. Interestingly, we found that H19 was upregulated in ADSC-induced chondrocytes compared to control ADSCs. H19 is a highly conserved gene that codes for an untranslated lncRNA. The gene itself consists of five exons separated by small introns. The transcript is a 2.3 kb capped, spliced, and polyadenylated RNA. As an imprinted gene, H19 is exclusively transcribed from the maternal allele and its expression level is regulated at the posttranscriptional level [37]. Numerous lncRNAs have been reported to be restricted to certain cell lineages, implying their potential function in the determination of cell fate [38]. Increasing evidence indicates that lncRNAs play important roles in MSC differentiation, especially osteoblast differentiation [39, 40]. However, there is a lack of data concerning the global expression and potential function of lncRNAs in the differentiation of human ADSCs into chondrocytes.

We found that the expression of H19 increased during ADSC differentiation into chondrocytes and that H19 was significantly involved in the process. Differentiation could be achieved simply by upregulating H19 in ASDCs. Usually, MSCs are induced to differentiate into different cell types by specific conditioned induction medium. We therefore propose that H19 is crucial for chondrocytic differentiation in ADSCs. It has been reported that H19 is abundantly expressed in the developing embryo, mainly in mesoderm- and endoderm-derived tissues; however, its expression is significantly decreased after birth [41]. Although it was discovered over two decades ago, its function has not been fully elucidated. Some studies have demonstrated that high expression of H19 is associated with cell proliferation, apoptosis, and metastasis in tumors [42, 43]. It has also been reported to be involved in tissue repair; for example, H19 can promote tendon regeneration by activating TGF-*β*1 signaling [44], and it plays an important role in diabetic wound healing as an indispensable regulator of vascular regeneration [45]. More importantly, H19 promotes fracture healing because of its positive effect on proliferation and its negative effect on apoptosis in osteoblasts and chondrocytes [46]. Consistently, in this study, H19 acted as a promoter in ADSC's differentiation into cartilage cells. These findings are potentially important for regenerative medicine.

To understand the underlying mechanism of H19 during chondrocyte differentiation in ADSCs, the KEGG database was applied to screen for DEGs. As a result, 276 DEGs were recognized and 113 genes were included in the pathway category analysis. As a result, fatty acid metabolism, the glycolysis/gluconeogenesis signaling pathway, and metabolism processes were inhibited. Biosynthesis processing, cell adhesion molecules, the chemokine signaling pathway, the RIG-I-like receptor signaling pathway, the osteoclast differentiation pathway, and immune system processes were upregulated. Based on this finding, ceRNA analysis showed that 22 miRNAs inhibiting H19 were downregulated, leading to an increase of 97 mRNAs that are the downstream targets of these miRNAs. It reported that STAT2 and JAK/STAT pathway (osteoclast differentiation pathway) were closely associated with cartilage relevant diseases, such as osteoarthritis (OA) cartilage or in human articular chondrocyte mechanotransduction [47], the bovine intervertebral disc [48], and rheumatoid arthritis (RA) [49]. We focused on the miRNAs and mRNAs involved in the osteoclast differentiation pathway, including miR-185-3p, miR-128-3p, miR-149-5p, miR-29c-3p, miR-195-5p, let-7c-5p, let-7b-5p, miR-324-3p, miR-205, miR-210-5p, STAT2, and IRF9, and examined their expression. As a result, STAT2 and IRF9 expressions were higher in the H19-overexpressing ADSC cell line (ADSC-H19) than in the control ADSC-pcDNA3.1 cells, while miR-185-3p, miR-128-3p, miR-149-5p, miR-195-5p, let-7c-5p, let-7b-5p, miR-324-3p, miR-205, and miR-210-5p were expressed at a lower level. However, the expression of miR-29c-3p showed no significant difference between ADSC-H19 cells and ADSC-pcDNA3.1 cells. To further confirm their regulatory role, we in turn detected related miRNAs and STAT2/IRF9 in the media-induced cartilage differentiation; the results indicated that STAT2 and IRF9 were obviously higher in the process, and all the miRNAs above were downregulated. But there is no statistically significant difference with miR-185-3p and miR-205 (Supplementary Figure 1). That may br because there are many molecules involved in chondrocytic differentiation of ADSCs, and H19 was only one of these functional roles in the process.

LncRNAs have been demonstrated to regulate the expression of some proteins at multiple levels, including epigenetic, transcriptional, and posttranslational [50, 51]. Given the well-known mechanisms of lncRNAs, the concept of ceRNA (competing endogenous RNA) was proposed by Tay et al. [52], which hold that lncRNAs, pseudogenes, circular RNAs, and mRNAs may impair the activities of miRNAs through sequestration, resulting in increased expression of miRNA target genes. Emerging evidence supports this hypothesis. For example, some protein-coding genes and their pseudogenes possess identical evolutionarily conserved miRNA binding sites in their 3′-untranslated regions, so that they may exert different, even opposite effects on gene expression by competing for miRNA binding [53]. This pattern was also verified by Franco-Zorrilla et al., who found that noncoding RNA interferon-*β* promoter stimulator 1 promotes phosphate metabolism (PHO) 2 protein by sequestering miR-399 and preventing its negative regulatory effect on PHO2 mRNA [54]. Given the above, we speculate that H19 regulates ADSC cartilage differentiation by competing for miRNAs that regulate the STAT2 pathway.

Application of ADSCs is a promising therapeutic strategy in various diseases, especially in regenerative medicine, and understanding their potential is critical. We examined their differentiation capacity toward cartilage cells, which could be the basis for treating bone- and joint-related diseases. However, the details of mechanism require much more work to be completely investigated, i.e., the interaction between H19 and miRNAs, and the STAT2/IRF9 as target of these miRNAs need further certification; moreover, the function of STAT2 and IRF9 remained to be identified at next-generation work.

## 5. Conclusions

H19 exerted its regulatory function during cartilage differentiation of ADSCs through competing miRNA regulation of STAT2.

## Data Availability

The authors declare that all the data supporting the findings in this study are available from the corresponding author through email in reasonable request.

## Supplementary Materials

Supplementary Materials Supplementary Figure 1: the expression of the related miRNAs and STAT2/IRF9 in the media-induced cartilage differentiation. (A) The expression of the related miRNAs was detected by qRT-PCR. (B) The mRNA levels of STAT2 and IRF9 in ADSCs and cartilage-induced ADSCs. (C) Protein levels of STAT2 in ADSCs and cartilage-induced ADSCs. (D) Protein levels of IRF9 in ADSCs and cartilage-induced ADSCs (^∗^ *P* < 0.05, ^∗∗^ *P* < 0.01, ^∗∗∗^ *P* < 0.001).
