# Supplementary figures and images for: Long Noncoding RNA H19 Participates in the Regulation of Adipose-Derived Stem Cells Cartilage Differentiation
Source: Stem Cells Int. 2019 May 5;2019:2139814. doi: 10.1155/2019/2139814 (PMC6525810; doi:10.1155/2019/2139814)

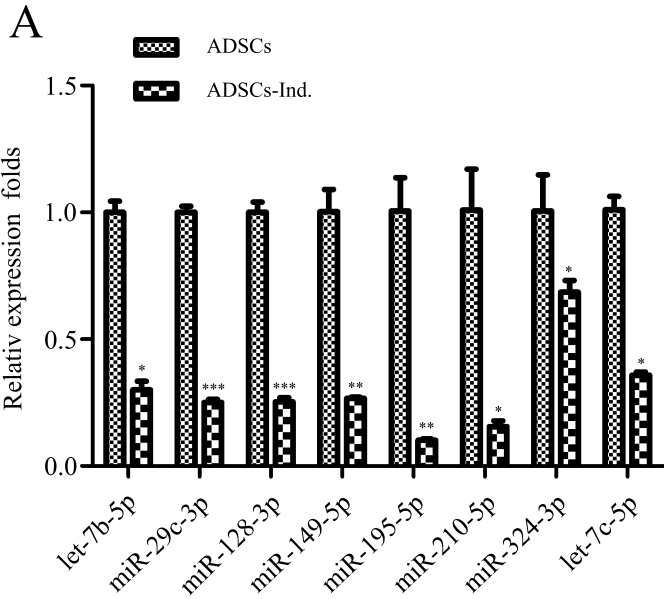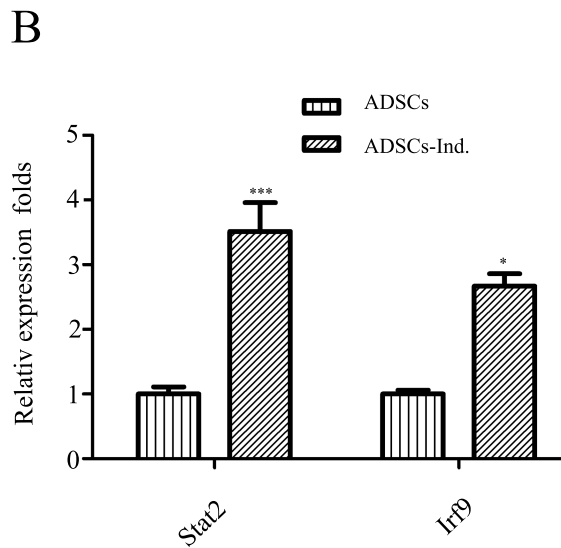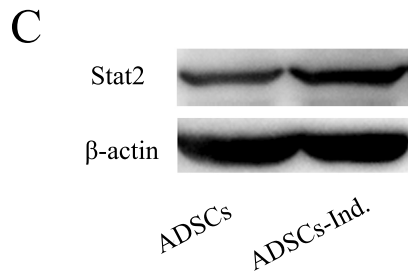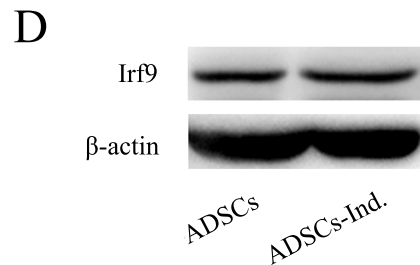

Supplement: Supplementary Materials — Supplementary Figure 1: the expression of the related miRNAs and STAT2/IRF9 in the media-induced cartilage differentiation. (A) The expression of the related miRNAs was detected by qRT-PCR. (B) The mRNA levels of STAT2 and IRF9 in ADSCs and cartilage-induced ADSCs. (C) Protein levels of STAT2 in ADSCs and cartilage-induced ADSCs. (D) Protein levels of IRF9 in ADSCs and cartilage-induced ADSCs (∗ P < 0.05, ∗∗ P < 0.01, ∗∗∗ P < 0.001). [file 2139814.f1.pdf]
